# An Exploratory Analysis of the Relationship between Cardiometabolic Risk Factors and Cognitive/Academic Performance among Adolescents

**DOI:** 10.1155/2015/520619

**Published:** 2015-06-07

**Authors:** Ting-Kuang Yeh, Ying-Chun Cho, Ting-Chi Yeh, Chung-Yi Hu, Li-Ching Lee, Chun-Yen Chang

**Affiliations:** ^1^Science Education Center and Graduate Institute of Science Education, National Taiwan Normal University, 88 Section 4, Ting-Chou Road, Taipei 116, Taiwan; ^2^Institute of Marine Environmental Science and Technology, National Taiwan Normal University, 88 Section 4, Ting-Chou Road, Taipei 116, Taiwan; ^3^Department of Earth Sciences, National Taiwan Normal University, 88 Section 4, Ting-Chou Road, Taipei 116, Taiwan; ^4^Department of Pediatrics, Mackay Memorial Hospital, 92 Section 2, Zhongshan N. Road, Taipei 104, Taiwan; ^5^Department of Clinical Laboratory Sciences and Medical Biotechnology, College of Medicine, National Taiwan University, No. 1, Section 1, Jen-Ai Road, Taipei 100, Taiwan

## Abstract

This exploratory study examines the relationship between cardiometabolic risk factors (blood pressure, waist circumference, BMI, and total cholesterol) and cognitive/academic performance. In this study, 1297 Taiwanese tenth-grade volunteers are recruited. Scores from the Basic Competency Test, an annual national competitive entrance examination, are used to evaluate academic performance. Cognitive abilities are accessed via the Multiple Aptitude Test Battery. The results indicate that systolic blood pressure is significantly, negatively associated with academic performance, both in male and female subjects. BMI and waist circumference are associated with verbal reasoning performance with an inverse U-shaped pattern, suggesting that both low and high BMI/waist circumference may be associated with lower verbal reasoning performance.

## 1. Introduction

Blood pressure, waist circumference, BMI, and serum cholesterol level are important predictors of the cardiovascular disease and metabolic syndrome. There is increasing interest in the effects of aforementioned cardiometabolic risk factors on human cognitive performance, mainly because the incidence of cardiovascular disease and metabolic syndrome has increased dramatically in adults, in adolescents, and even more remarkably in children over the past few decades [1, 2]. Furthermore, human cognitive performances are usually affected by physiology activity and environment factors or vice versa. Understanding the impact of physical activity in cognition behavior could provide information for development of promising prevention and treatment strategies.

The correlation between blood pressure and cognitive impairment in patients has already been established [3]. Evidence revealed that both hypertension and hypotension play a part in the development and progression of cognitive impairment and dementia. Compared with normotensive population, patients with hypertension showed poorer performance on tasks of attention, learning and memory, executive functions, visuospatial skills, psychomotor abilities, and perceptual skills [4, 5]. Subjects with hypotension performed poorer on tasks of verbal short-term memory, mental arithmetic tasks, executive function, selective attention, and verbal recognition tasks [6].

Both obesity and underweight have been suggested as risk factors for dementia and worse cognitive performance. Studies in older adults have found an association between obesity and poor cognitive performance [7] and between underweight and poor cognitive performance [8], in several population-based investigations [9]. Several studies reported that long-term obesity and long-term underweight in adulthood are associated with lower cognitive scores in midlife [10, 11]. Meta-analysis data showed there was a significant U-shaped association between BMI and dementia, with dementia risk being increased for obesity and underweight [12].

Relationship between hyperlipidemia and cognitive functioning is currently a matter of debate. Some studies have found a correlation between hyperlipidemia and both vascular dementia and Alzheimer's disease in cross-national surveys, whereas other studies have not [13, 14]. In a sample of 1894 cohorts, Elias et al. [15] found that lower total cholesterol levels are associated with poorer performance on cognitive measures. However, Anstey et al. [16] conducted a systematic review and concluded that higher cholesterol is a risk factor for dementia and cognitive decline.

Literature, over the past decade, suggests that the hyper/hypotension, obesity, underweight, and hyperlipidemia are associated with cognitive decline in patients; however, the mechanism(s) by which abnormal blood pressure, body weight, and dyslipidemia affect human cognition remains unexplored. Studies on the relationship between cardiometabolic risk factors and cognitive function in normal population could provide researchers with evidence to establish relative mechanisms [17]. Previous exploratory studies with sampling from general population appear to be mainly based on evidence from adult, yet rarely in young persons. Besides, these studies revealed inconsistent results in investigating the association between cardiometabolic risk factors and cognitive performance. For blood pressure instance, cross-sectional studies have shown different relationship between hypertension and cognitive abilities in middle-aged and elderly adults, including positive linear, inverse relation, *J*-curve, *U*-curve, or no associations. A limited number of studies have provided evidence that excess weight in adolescents will alter cognitive functions selectively. For example, Alosco et al. [18] suggest that obesity in children and adolescents is associated with decreased volume of frontal and limbic cerebral gray matter regions. Li et al. [19] observed 2519 children and adolescents (aged 8–16) and reported that increased body weight is independently associated with decreased visual-spatial organization and general mental ability. A similar finding also reported that extremely obese adolescents exhibited deficits in many cognitive domains, including impairment in attention and executive functions [20]. However, Gunstad et al. [21] indicated that underweight might be a risk factor for reduced memory performance in females. Datar et al. [22] concluded that worse performances in cognitive task scores by overweight status were explained by parental education and home environment rather than overweight status per se. To our best knowledge, few studies have been conducted to explore the correlation between lipidemia and cognitive performance, especially in the normal adolescent population.

As mentioned by Lyngdoh et al. [17], data from randomized controlled trials could provide convincing evidence to explore the mechanisms of the impact of cardiometabolic risk factors on cognitive function. Studies on the relationship between cardiometabolic risk factors and cognitive function in adolescent are rare. As cognitive abilities usually impact academic performance significantly, education researchers may be concerned with the issue of whether cardiovascular factors are associated with cognitive abilities in adolescents. Few studies have investigated the correlation among cardiometabolic risk factors, cognitive abilities, and academic performance. This study attempted to fill this gap. We hypothesized that cardiometabolic risk factors are associated with learners' cognitive abilities and academic performance. It is our hope that this research will clarify future directions for the investigation of behavioral mechanisms for researchers in various fields.

## 2. Methodology

### 2.1. Participants

In Taiwan, students who wish to attend secondary-level education, after junior high school, must take the annual Basic Competency Test (BCT), a national standardized test that measures educational achievement, in order to enroll in a senior high school. In general, more than 300,000 examinees (equal to 95% of this age group) take the BCT each year. In order to select a nationally representative sample in terms of academic achievement, three public senior high schools were selected (one in southern Taiwan, one in the middle of Taiwan, and the other in northern Taiwan). A total of 1297 tenth-grade volunteers, 464 male and 833 female, all of whom being Han Chinese, were recruited for this study. The mean age of the subjects was 16.8 years (SD, 0.3; age range: 16-17 years). Statistical analysis of goodness-of-fit showed that when the scores of these senior high-school students were combined, they gave a good representation (simulation) of the national probability distribution (*χ* ^2^ = 21). The volunteers and their parents were all informed explicitly about the plan, protocol, and procedure for the study, and written consent was obtained prior to the study being performed. This study was approved by the institutional review board of the National Taiwan University Hospital.

### 2.2. Academic Achievement Scores

The Basic Competency Test (BCT) consists of a 2-day written test program that covers six subjects: Chinese, English, mathematics, science, social science, and writing. Subtests within the BCT consist of 34–63 multiple choice questions, which include testlets and simple items. Examinees have 70 minutes to complete each subtest. Item response theory (IRT) models [23, 24] are used to convert the examinees' raw scores to a score scale (for the writing test the range is 0–10; for the other subtests the range is 0–60).

### 2.3. Cognitive Abilities Assessment

Cognitive abilities were assessed via the Multiple Aptitude Test Battery (MAT) [25, 26], which is commonly used, thoroughly standardized, and suitable for assessment of the diversity of cognitive abilities in adolescents. The Chinese version of MAT was revised from the Differential Aptitude Tests (DAT, [25]), aimed to provide an integrated, scientific, and well-standardized procedure for measuring the cognitive abilities of Taiwanese adolescents. In theory, aptitude testing can provide more accurate predictions for learning and working performance than traditional intelligence tests [27]. The MAT consists of eight subtests: verbal reasoning, numerical ability, mechanical reasoning, perceptual speed and accuracy, space relations, abstract reasoning, verbal comprehension, and grammar and language usage. This test contains a total of 496 items and takes about 80 minutes for administration. Mao and Lu [26] reported the internal consistency reliability of the MAT ranged from 0.5 to 0.9.

### 2.4. Anthropometric and Biochemical Parameters

All the volunteers underwent clinical assessments, including the measurement of systolic blood pressure (SBP) and diastolic blood pressure (DBP), which were measured in accordance with Joint National Committee VII guidelines. Measurements were performed in triplicate with 2-minute intervals of quiet seated rest, and the average values were calculated.

Body mass index (BMI) was calculated as weight (kilogram) divided by height (meters) squared. Weight was measured with light clothing, using a precision electronic scale, recorded to the nearest 0.1 kg, and height was measured without shoes, with a fixed stadiometer, recorded to the nearest cm. A venous blood sample was taken from each participant after 12 hours of fasting to measure total serum cholesterol level.

### 2.5. Statistical Analysis

Two-tailed *t*-test analyses were performed on the results of the variables, including cardiometabolic risk factors, cognitive abilities, and academic performance, between males and females. Pearson correlations were conducted as an exploratory analysis to examine bivariate associations between the variables. The level of confidence was set at the 0.05 significance level. Permutation tests (with *N* = 1000 randomizations) [28] were performed to correct for multiple comparisons. To meet contemporary calls for improvement in the interpretation and reporting of quantitative research [29], we have reported the practical significance (effect size) along with each statistical significance test. In a larger sample size, it is more likely to observe a statistical significance, even if there is little practical effect. According to Cohen's rough characterization, for Pearson correlation analysis, *r* = 0.1 is deemed to be a small effect size, *r* = 0.3 a medium effect size, and *r* = 0.5 a large effect size. We also tried to test the linear/curvilinear relationships between variables. Tests of the assumptions and inferential statistical analyses were performed using SPSS version 18.0 (Chicago, IL, USA).

## 3. Result

Demographic and clinical characteristics of the participants are outlined in Table 1. Some differences between males and females were observed. Male subjects in this study perform better in BCT, mechanical reasoning, space relations, and abstract reasoning subtests, whereas female subjects perform better in verbal comprehension and grammar and language usage subtests. Male subjects had higher mean SBP BMI and waist circumference.

In this study, we performed analyses by sex separately because cardiometabolic factors and cognitive abilities were different between males and females. As shown in Tables 2 and 3, bivariate correlations revealed that systolic blood pressure was significantly negatively correlated with academic performance (BCT), in both males (*r* = 0.21,  *P* < 0.001, small to medium effect size) and females (*r* = 0.12, *P* < 0.01, small effect size). After 1000 permutation tests were performed, the results were not altered (males: mean of *r* = 0.21, SD of *r* = 0.03; females: mean of *r* = 0.12, SD of *r* = 0.03). No significant associations in the other cardiometabolic risk factors and academic performance were observed. Academic achievement was positively associated with cognitive abilities.

With regard to the cognitive performance, BMI and waist circumference (WC) were positively associated with verbal reasoning, both in males (BMI: *r* = 0.13, *P* < 0.01; WC: *r* = 0.13, *P* < 0.01) and in females (BMI: *r* = 0.12, *P* < 0.01; WC: *r* = 0.10, *P* < 0.01), with small effect size. After 1000 permutation tests were performed, the results were not altered (males: BMI *r* = 0.13, SD = 0.03; WC: *r* = 0.13, SD = 0.04; females: BMI *r* = 0.12, SD = 0.03; WC: *r* = 0.10, SD = 0.03). No consistent evidence of an association between cognitive abilities and other cardiometabolic risk factors was observed.

Systolic blood pressure was positively associated with diastolic blood pressure (males: *r* = 0.42; females: *r* = 0.58, permutation tests: males: *r* = 0.42, SD = 0.01; females: *r* = 0.57, SD = 0.02), BMI (males: *r* = 0.28; females: *r* = 0.29, permutation tests: males: *r* = 0.28, SD = 0.03; females: *r* = 0.29, SD = 0.02), and waist circumference (males: *r* = 0.32; females: *r* = 0.20, permutation tests: males: *r* = 0.32, SD = 0.03; females: *r* = 0.21, SD = 0.03). BMI was highly correlated with waist circumference (males: *r* = 0.93; females: *r* = 0.89; permutation tests: males: *r* = 0.93, SD = 0.01; females: *r* = 0.89, SD = 0.01).

We further tried to test the linear/curvilinear relationships between systolic blood pressure and BCT, BMI and verbal reasoning, and waist circumference and verbal reasoning outcomes. The result suggested that the linear specification fitted the data (male: *R* ^2^ = 0.04, *P* < 0.01; female: *R* ^2^ = 0.01, *P* = 0.01) better than the curvilinear when testing the relationship between systolic blood pressure and BCT. When testing the relationship between BMI and verbal reasoning, both inverse *U* curve and linear specification were fitting the data significantly, but the inverse *U* curve had better explanation rate (for inverse *U*-curve shaped male: *R* ^2^ = 0.02, *P* < 0.01; female: *R* ^2^ = 0.02, *P* < 0.01; for linear specification: *R* ^2^ = 0.015, *P* < 0.05; female: *R* ^2^ = 0.015, *P* < 0.01). Inverse *U* curve was also shown to fit the data better than linear specification when testing the relationship between waist circumference and verbal reasoning outcome (for inverse *U*-curve shaped male: *R* ^2^ = 0.02, *P* < 0.01; female: *R* ^2^ = 0.02, *P* < 0.01; for linear specification: *R* ^2^ = 0.018, *P* < 0.05; female: *R* ^2^ = 0.01, *P* > 0.05).

## 4. Discussion

The factors that impact academic achievement of substantial importance are not yet understood, nor explored fully [30]. As shown in Table 2, the results of this study indicated that systolic blood pressure was negatively associated with subjects' BCT scores, in both males and females. Since no significant associations between systolic blood pressure and cognitive abilities were observed, the evidence of the current study revealed that cognitive abilities did not play important moderator or mediator roles between systolic blood pressure and BCT. In other words, the cognitive abilities did not involve in the major mechanism of systolic blood pressure on students' BCT performance (and vice versa).

It is possible that the academic performance of students in the BCT might be influenced substantially by the beneficial emotional effects of the blood pressure. As mentioned above, the annual BCT in Taiwan is a high-stake test that is used to determine which students can enter senior high school and which school they will be assigned to. Therefore, students are subjected to intense pressure due to the competition and long-term preparation for this examination and the impact that it will have on their future educational opportunities. A number of studies indicated higher blood pressure was associated with anger, anxiety, and depression [31–35]. Ewart and Kolodner [33] reported that trait anger and negative effect were accompanied by higher blood pressure in adolescents. Chen and his colleagues [32] also found that depression was significantly associated with undetected hypertension. At the educational level, previous studies have reported that affective factors, such as testing and learning anxiety, the negative impact of emotional vulnerability, and academic stress, can have a major influence on academic performance [36–38]. In conclusion, the significant correlation between systolic blood pressure and students' BCT performance might result from the influence of stress and emotional vulnerability. However, due to the lack of measurement of the participants' emotion factors, the current study cannot establish the relationship among systolic blood pressure, emotion factors, and BCT performance via regression analysis [39, 40]. In subsequent studies, it would be of interest to perform rigorous hypothesis testing regarding this issue by recording and analyzing more complete information.

The results of this study also revealed that BMI and waist circumference were associated with verbal reasoning with small effect size, in both males and females. The inverse *U* curve fitted the relationship data better than linear specification, suggesting that both low and high BMI/waist circumference may be associated with lower verbal reasoning performance. Although the role of extreme obesity and underweight in cognitive function in adults has been reported [7, 8, 10, 12], to our best knowledge, few studies have examined the association between BMI/waist circumference and cognitive function in adolescents. Li et al. [19] reported that increased body weight is associated with decreased general mental ability in adolescents, whereas Gunstad et al. [21] indicated that underweight might be a risk factor for reduced memory performance. A recent study showed that adolescents with BMI in the normal range performed better than their peers in the underweight and obese weight ranges [41]. These studies provide evidence that both obesity and underweight may be associated with worse cognitive abilities. Our result revealed the association between BMI and verbal reasoning was consistent with those of the meta-analysis study conducted by Beydoun et al. [12], showing a significant U-shaped association between BMI and dementia.

In this study, no significant associations in the other cardiometabolic risk factors and cognitive abilities/academic performance were observed. With respect to the blood pressure, previous exploratory studies with sampling from general population revealed inconsistent results in investigating the association between blood pressure and cognitive performance. The finding of this study parallels recent evidence, in large adolescent populations, that there is no consistent association between BP and cognitive performance [17, 42]. It is important to note that Lande et al. [42] reported children with elevated systolic BP (SBP ≧ 90th percentile, *n* = 288) had lower average scores compared with normotensive children (SBP < 90th percentile, *n* = 4789) for digit span, block design, and mathematics in a nationally representative sample of 5077 children aged 6–16 years in the US (NHANES III). From a statistical perspective, it is quite common to observe a statistical significance with a large sample size, even if there is little practical effect. We found there was no significant effect between SBP and cognitive abilities after calculating the effect size from the result of NHANES III data (Cohen's *d* of block design, digit span, and mathematics outcomes are 0.13, 0.15, and 0.17, resp.; according to Cohen's rough characterization (1988, pp. 284–288), *d* = 0.2 is deemed to be a small effect size, 0.5 a medium effect size, and 0.8 a large effect size). Our findings are partly in agreement with the Lande et al. [42] study (NHANES III) with nearly a small effect size for the negative association between SBP and numerical performance.

Relationships between cholesterol level and cognitive functioning have been less consistent. Few studies have measured the correlation between cholesterol level and cognitive abilities/academic performance in the general population. The result of this study shows no significant association between cholesterol level and cognitive abilities. A possible reason is that mild change in cardiometabolic risk factors may not be enough to trigger manifestations of cognitive decline [17]. A complex and dynamic network exists among cardiometabolic risk factors, cognitive abilities, and academic achievement. We have only examined the correlation of some variables. It is possible that associations between cardiometabolic risk factor and cognitive abilities/academic performance could be influenced by accumulation/interactions of emotion, cultural difference, physical activity, social-economic level, dietary habits, genes regulation, and so forth. At the same time, we must also reflect on the limitations of this study and some improvements that could be made. A potential limitation is that most of the participants were relatively physically healthy. Since cardiometabolic risk factors are affected by the interaction of multiple factors, it will be more difficult to attain statistical/practical significance with respect to the association between physiological activity and cognitive/academic performance. However, since it is more difficult to observe a statistical significance with a normal population, statistically significant results with only small to medium effect sizes in this study might be useful for researchers in their further exploration of relevant mechanisms at the neural/molecular levels.

During the learning process, students who do not perform as well academically as others due to poor cognitive abilities or emotional self-control show a decreased willingness to learn. The integration of physiology and psychology data might have the potential advantages to understand learning mechanisms and then find strategies for promoting learning. For example, the evaluating of physiology and serum biochemistry (such as cardiometabolic risk factors) of students could give strategic educationists an understanding of the possible change of cognitive abilities. They could then give priority to provide appropriate learning environment and monitor the cognitions and emotions of the student in learning. In this way, the student's interest in learning and achievement could be increased. We envisage that the integration of cognition and physiology study will provide researchers in different fields with evidence that can be used to untangle the complicated relationships/mechanisms of behavior.

## Figures and Tables

**Table 1:** Distribution of selected characteristics among participants.

	Male	Female	*P*
Age (yrs)	16.8 (0.32)^*^	16.8 (0.30)	
Academic performance			
BCT	245.9 (19.69)	235.8 (20.61)	<0.01
Cognitive abilities			
Verbal reasoning	21.5 (5.31)	21.9 (5.16)	
Numerical ability	11.1 (3.56)	10.90 (3.37)	
Mechanical reasoning	14.3 (3.91)	13.3 (3.46)	<0.01
Space relations	17.1 (4.49)	15.4 (4.49)	<0.01
Abstract reasoning	20.2 (4.81)	19.1 (4.95)	<0.01
Verbal comprehension	21.5 (6.14)	22.2 (5.91)	<0.05
Grammar and language usage	17.9 (5.04)	18.5 (5.00)	<0.05
Perceptual speed and accuracy	67.2 (21.33)	68.1 (20.19)	
Cardiometabolic factors			
Systolic BP (mmHg)	124.4 (12.27)	113.93 (11.36)	<0.01
SBP range	88–162	70–151	
Diastolic BP (mmHg)	70.8 (9.32)	70.8 (7.97)	
DBP range	47–100	51–96	
Cholesterol (mg/dL)	172.3 (35.21)	171.9 (30.04)	
Range	111–268	99–258	
BMI (kg/m^2^)	21.6 (3.77)	20.3 (3.21)	<0.01
BMI range	15.6–33.5	14.6–33.6	
Waist circumference (cm)	73.90 (10.23)	66.17 (7.81)	<0.01
WC range	58–108	50–99	

^*^Mean (SD).

**Table 2:** Intercorrelations between study variables in female subjects.

	1	2	3	4	5	6	7	8	9	10	11	12
(1) BCT (academic performance)	—											
Cognitive abilities												
(2) Verbal reasoning	0.26^*^	—										
(3) Numerical ability	0.35^**^	0.25^**^	—									
(4) Mechanical reasoning	0.21^*^	0.32^**^	0.29^**^	—								
(5) Space relations	0.13^*^	0.26^**^	0.25^**^	0.33^**^	—							
(6) Abstract reasoning	0.12^*^	0.28^**^	0.31^**^	0.35^**^	0.46^**^	—						
(7) Perceptual speed and accuracy	0.22^*^	0.36^**^	0.27^**^	0.38^**^	0.20^**^	0.36^**^	—					
Cardiovascular factors												
(8) Systolic BP	−0.12^*^	0.03	−0.06	−0.06	−0.03	0.04	0.06	—				
(9) Diastolic BP	−0.06	0.01	0.03	−0.07	−0.01	0.04	0.04	0.58^***^	—			
(10) BMI	−0.04	0.12^*^	0.04	0.01	0.03	0.04	0.04	0.29^**^	0.18^*^	—		
(11) Waist circumference	−0.08	0.10^*^	0.06	0.10^*^	−0.01	0.08	−0.01	0.20^**^	0.11^*^	−0.89^***^	—	
(12) Cholesterol	−0.03	−0.01	−0.01	−0.01	0.02	0.03	0.07	0.03	0.07	−0.01	0.10^*^	—

^*^ *P* < 0.05; ^**^ *P* < 0.01.

**Table 3:** Intercorrelations between study variables in male subjects.

	1	2	3	4	5	6	7	8	9	10	11	12
(1) BCT (academic performance)	—											
Cognitive abilities												
(2) Verbal reasoning	0.10^*^	—										
(3) Numerical ability	0.01	0.44^**^	—									
(4) Mechanical reasoning	0.04	0.40^**^	0.39^**^	—								
(5) Space relations	−0.04	0.47^**^	0.35^**^	0.39^**^	—							
(6) Abstract reasoning	0.06	0.42^**^	0.38^**^	0.35^**^	0.45^**^	—						
(7) Perceptual speed and accuracy	0.11	0.41^**^	0.48^**^	0.42^**^	0.44^*^	0.46^**^	—					
Cardiovascular factors												
(8) Systolic BP	−0.21^*^	−0.02	0.02	−0.03	0.04	0.04	−0.02	—				
(9) Diastolic BP	−0.03	−0.04	0.02	−0.03	0.01	0.04	0.05	0.42^**^	—			
(10) BMI	−0.09	0.13^*^	0.07	0.05	0.07	0.02	0.05	0.28^**^	0.08	—		
(11) Waist circumference	−0.02	0.13^*^	0.05	0.05	0.07	0.01	0.04	0.31^**^	0.11^*^	0.93^***^	—	
(12) Cholesterol	−0.07	0.07	0.06	0.03	0.04	0.06	0.05	0.06	0.11^*^	0.26^**^	0.28^**^	—

^*^ *P* < 0.05; ^**^ *P* < 0.01.
